# 

**Published:** 1905-05-13

**Authors:** 


### NOTICE TO CORRESPONDENTS.

All MSS., Letters, Books for Review, and other matters intended for the Editor should be addressed THE EDITOR, "THE HOSPITAL," THE HOSPITAL BUILDINGS, 28 & 29 SOUTHAMPTON STREET, STRAND, W.C.

The Editor cannot undertake to return rejected MS., even when accompanied by stamped directed envelope.

### Notice to Advertisers and Subscribers.

All Advertisements, Orders for Copies of THE HOSPITAL, and Business Communications should be addressed to **THE MANAGER (Not to the Editor),** THE HOSPITAL, 28 & 29 Southampton Street, Strand, London, W.C.

The Cost of Subscription, post free, is at follows:—Per week, 3½d.; per quarter, 3s. 3d.; per half-year, 6s. 6d.; per annum, 13s. Foreign and Colonial:—Per annum, 19s. 6d., payable, in all cases, in advance.

**NOTICE.—Vol. XXXVII. of "The Hospital" October 1904 to March 1905), in handsome cloth binding, will shortly be on sale at the office of this Journal, price 10s. 6d. Cases for binding the Numbers contained in this Volume 2s. Index to Vol. XXXVII., 3½d., post free.**

**The Monthly Part for May is now ready. Price 1s., by post, 1s. 3d.**

### BOOKS RECEIVED.

A. Constable and Co., Limited.

"Surgical Case-Book Charts for Rectal Diseases." By C. H. Leaf, F.R.C.S.

W. Blackwood and Sons.

"Saints and Savages." By Robert Lamb, M.A.

J. and A. Churchill.

"Practice of Midwifery for Nurses." Second edition. By H. Jellett, M.D.

"A System of Clinical Medicine." Two vols. By T. D. Savell, M.D.

Baillière, Tindall and Cox.

"Lectures on Clinical Surgery." By H. C. Hinder, M.B., M.Ch.

Stevens and Sons, Limited.

"Income Tax Tables." By G. H. Whybrow.

John Wright and Co., Bristol.

"Poisonous Plants of all Countries." By A. Bernhard Smith.

"First Aid to the Injured and Sick." (Third Edition.) By F. J. Warwick, M.B., and Tunstall, M.D., F.R.C.S.

### Medical Appointments.

**J. M. Ambrose**, L.R.C.P.& S.Edin., Certifying Surgeon under the Factory and Workshop Act for the Ardagh District of the County of Limerick. **A. M. Barford**, M.D.Brux., D.P.H.R.C.P.S. Edin., L.R.C.P.Lond., M.R.C.S., Physician to the Chichester General Infirmary. **A. Brook Batley**, L.R.C.P.Lond., M.R.C.S., Medical Officer to the Christchurch (Hants) Union Workhouse and Cottage Homes. **Francis Thomas Bond**, M.D.Lond., M.R.C.S., F.R.S.Edin., Medical Officer of Health for the Tetbury Urban District Council and Medical Officer of Health of the Chippenham Urban District. **Edgar Chatterton**, M.R.C.S.Eng., L.R.C.P.Lond., Pathologist to the Western Ophthalmic Hospital, Marylebone Road, W. **Joseph Basil Cook**, M.D., B.Ch.Vict., M.R.C.S., L.R.C.P.Lond., Assistant Medical Superintendent to the Kensington Infirmary, Marloes Road, W. **W. I. Hancock**, F.R.C.S.Eng., Surgeon to the Ophthalmic Department of the Bolingbroke Hospital, Wandsworth Common, S.W. **Charles John Harnett**, M.D.Lond., M.R.C.S., L.R.C.P.Lond., Honorary Visiting Surgeon to the Royal Sea Bathing Hospital, Margate. **Frank Hewkley**, M.B.Durh., Physician to the St. Luke's Medical Mission. **William Moore Hope**, M.R.C.S., L.S.A., D.P.H.Eng., Medical Officer of Health of the City of Gloucester. **Robert Jaques**, F.R.C.S.Eng., House Surgeon to the Royal Eye Hospital, Southwark, S.E. **C. I. Kelly**, L.S.A., Certifying Surgeon under the Factory and Workshop Act for the Portumna District of the county of Galway. **Thomas Curtis Leman**, M.D.Brux., M.R.C.S., L.R.C.P.Lond., Medical Officer of Health by the Chipping Sodbury (Gloucestershire) District Council. **J. L. Lock**, M.B., B.S.Cantab., Certifying Surgeon under the Factory and Workshop Act for the Uxbridge District of the County of Middlesex and for the Denham District of the County of Buckingham. **W. MacCarthy**, M.B., B.Ch., B.A.O.R.U.I., Certifying Surgeon under the Factory and Workshop Act for the Abbeyfeale District of the County of Limerick. **R. E. Moriarty**, L.R.C.P.&S.Edin., L.F.P.S.Glasg., Certifying Surgeon under the Factory and Workshop Act for the Sherburn District of the County of York. **James Mudge**, M.R.C.S., L.R.C.P.Edin., L.S.A., Medical Officer of Health of the West Penwith (Cornwall) Rural District. **D'Arcy Power**, F.R.C.S.Eng., Surgeon to the Bolingbroke Hospital, Wandsworth Common, S.W. **Charles Ryall**, F.R.C.S.Eng., Assistant Surgeon to the Bolingbroke Hospital, Wandsworth Common, S.W. **S. R. Schofield**, M.B., Clinical Assistant to the Chelsea Hospital for Women. **Arnold Edward Soden**, M.R.C.S., L.R.C.P.Lond., Deputy Public Vaccinator for the Second District St. Columb Major (Cornwall) Union. **William Sykes**, M.D., F.S.A., Surgeon and Agent to Admiralty and Coastguard at Paignton, S. Devon, and Medical Examiner to Prudential Assurance Company for Paignton and District. **Mark Ronald Taylor**, L.R.C.P.Lond., M.R.C.S., Admiralty Surgeon and Agent at Porthleven and Gunwalloe, Cornwall. **H. Campbell Thomson**, M.D.Lond., F.R.C.P.Lond., Physician to the Bolingbroke Hospital, Wandsworth Common, S.W. **R. C. Twigg**, M.B., B.Ch., B.A.O., Anæsthetist to the Bolingbroke Hospital, Wandsworth Common, S.W. **Charles Arthur Wigan**, M.D.Durh., M.R.C.S., L.S.A., Chairman of the Portishead (Somerset) District Council. **William Wright**, M.B., B.C., D.P.H.Camb., Assistant Medical Officer of Health of Glasgow.

### The Nurse's Library.

**NURSING: Its Theory and Practice.**
Being a complete Text-book of Medical, Surgical, and Monthly Nursing.
By PERCY G. LEWIS, M.D. **3s. 6d.** post free.

**THE NURSE'S PRONOUNCING DICTIONARY OF MEDICAL TERMS AND NURSING TREATMENT.**
By HONNOR MORTEN.
Fifth Edition, Edited and Revised by MARY I. BURDETT.
**2s.** post free.

**MODERN NURSING OF CONSUMPTION.**

By Dr. JANE H. WALKER. **1s.** net, **1s. 2d.** post free.

**ART OF MASSAGE.**
By A. CREIGHTON HALE.
**6s.** post free.

**ELEMENTARY TREATISE ON THE LIGHT TREATMENT FOR NURSES.**
By JAMES H. SEQUEIRA, M.D. Lond.
**2s. 6d.** net, **2s. 9d.** post free.

**THE EXAMINATION OF THE URINE.**

By J. K. WATSON, M.D.

**1s.** net, **1s. 1d.** post free.

**POCKET BOOK OF TEMPERATURE CHARTS,**
containing 25 Charts,
6d. net, 7d. post free.

**TEMPERATURE CHARTS, Morning, Evening, and 4-hourly Charts.**

25 - - 1s. post free.

50 - - 1s. 6d. "

### The Nursing Mirror.
The Nurse's Own Paper.
**Price ONE PENNY Weekly.**
Of all Booksellers and Newsagents, post direct, or from the Office.

*Write for Subscription Rates to*
THE SCIENTIFIC PRESS, Ltd.,
28 & 29 Southampton Street, Strand,
London, W.C.

**A CASE BOOK FOR PRIVATE NURSING FOR NIGHT AND DAY REPORTS.**

Large size,

3d. net, 4½d. post free.

### For Midwives.

**A COMPLETE HANDBOOK OF MIDWIFERY.**
By J. K. WATSON, M.D. Edin., Author of "A Handbook for Nurses."
Crown 8vo. over 350 pp., profusely Illustrated with 143 Diagrams. **6s.** net, **6s. 4d.** post free.

**NURSING NOTES ON MIDWIFERY AND GYNÆCOLOGY.**

By Sister ROSS, Rotunda Hospital, Dublin.

Demy 16mo. cloth, **2s.** net, **2s. 1d.** post free.

(Suitable size for apron pocket.)

**CLEANLINESS IN CHILDREN.**
By ROSE PETTY, Associate of the Sanitary Institute.
Crown 8vo. paper cover, **2d.** post free.

**A PRACTICAL HANDBOOK OF MIDWIFERY.**
By FRANCIS W. HAULTAIN, M.D.
New and Revised Edition. 18mo. profusely Illustrated with Original Cuts, Tables, &c., handsomely bound in leather, **6s.** net, **6s. 3d.** post free.

**HOW TO BECOME A CERTIFIED MIDWIFE.**
By E. L. C. APPEL, M.B., B.S., B.Sc. Lond.
Crown 8vo. bound limp cloth, **1s.** net, **1s. 1d.** post free.

**THE MOTHER'S HELP AND GUIDE TO THE DOMESTIC MANAGEMENT OF HER CHILDREN.**
By P. MURRAY BRAIDWOOD, M.D.
Second Edition, with Addenda, crown 8vo. cloth,
**2s.** post free.

**The Scientific Press, Ltd.,** Publishers of all classes of Nursing Text-Books, Case-Books, Charts, &c.,

28 & 29 SOUTHAMPTON STREET, STRAND, LONDON, W.C.

